# Enhancement of the Solubility and Dissolution Profile of Rivaroxaban by the Antisolvent Precipitation Technique: A Promising Approach

**DOI:** 10.3390/polym18091134

**Published:** 2026-05-05

**Authors:** Claudia Maria Benga, Emma Adriana Ozon, Adina Magdalena Musuc, Valentina Anuța, Iulian Sârbu, Vasile-Adrian Surdu, Florina Teodorescu, Adriana Rusu, Lăcrămioara Popa, Mihaela Violeta Ghica, Abhay Chandak, Cristina Elena Dinu Pîrvu

**Affiliations:** 1 Faculty of Pharmacy, University of Medicine and Pharmacy “Carol Davila”, 6 Traian Vuia Street, 020945 Bucharest, Romania; claudia-maria.benga@drd.umfcd.ro (C.M.B.); emma.budura@umfcd.ro (E.A.O.); lacramioara.popa@umfcd.ro (L.P.); mihaela.ghica@umfcd.ro (M.V.G.); cristina.dinu@umfcd.ro (C.E.D.P.); 2 Institute of Physical Chemistry—Ilie Murgulescu, Romanian Academy, 202 Spl. Independentei, 060021 Bucharest, Romania; fteodorescu@icf.ro (F.T.); arusu@icf.ro (A.R.); 3 Faculty of Pharmacy, “Titu Maiorescu” University, 004051 Bucharest, Romania; iulian.sarbu@prof.utm.ro; 4 Department of Materials Science, Faculty of Materials Science and Engineering, Transilvania University of Brasov, 29 Eroilor Blvd., 500036 Brasov, Romania; vasile.surdu@unitbv.ro; 5 Zentiva Group, U Kabelovny 529/16, Dolní Měcholupy, 102 00 Prague, Czech Republic; abhaykumar.chandak@zentiva.com

**Keywords:** rivaroxaban, antisolvent precipitation, oral bioavailability, nanosuspensions, dissolution profile, Poloxamer 188

## Abstract

The development of new pharmaceutical forms with high solubility and enhanced bioavailability currently represents a significant challenge in the pharmaceutical industry. Currently, methods are still being explored to improve the oral bioavailability of Rivaroxaban, estimated to be 60%, due to its low solubility. To address these challenges, this study uses the antisolvent precipitation method to obtain three nanosuspensions of rivaroxaban (RIV), using Poloxamer 188 (P188) and hydroxypropyl methylcellulose (HPMC) by varying their concentrations (1:1:1, 1:1:2, and 1:2:1 molar ratios). The RIV nanosuspensions were characterized by Fourier transform infrared spectroscopy (FT-IR), X-ray powder diffraction (XRD), scanning electron microscopy (SEM), and thermogravimetric analysis (TGA). The antisolvent precipitation method led to the successful formulation of the three RIV nanosuspensions. Afterward, the formulated tablets containing dry RIV nanosuspensions were pharmaceutically characterized. RIV-P188-HPMC (1:1:1) and RIV-P188-HPMC (1:2:1) dry nanosuspensions demonstrated a uniform flow, and they were subsequently analyzed to establish the in vitro dissolution profile. The nanosuspension formulation with a higher content of P188 showed superior performance. Overall‚ the results of this study show that the antisolvent precipitation method in the presence of different amounts of HPMC and P188 is very efficient in increasing the dissolution rate of rivaroxaban to achieve its better efficiency.

## 1. Introduction

Recently, there has been intensive research to discover new potential candidates with excellent binding capacity for targeting receptors. Several modern methods have been developed for the creation of innovative drugs, such as genomic and proteomic approaches, cheminformatics, bioinformatics, systems biology, phenotypic, virtual, and natural product screening [1]. In general, the insolubility, variable and low bioavailability of active pharmaceutical forms and consequently of drugs, are determined by a series of physical and chemical factors such as few hydrogen bonds, a highly crystalline and stable network, a large molecular size, and high lipophilicity [2]. The reduced bioavailability of drugs due to their low solubility in water or lipids leads to a reduction in the drug concentration below the therapeutic level in the target tissue and, consequently, to its ineffectiveness [3]. Since 1897, Noyes A.A. and Whitney W.R. developed the Noyes–Whitney integral equation to characterize the dissolution profile of drugs in different media, which demonstrated that the bioavailability and solubility of drugs increase by reducing the particle size, which leads to an increase in surface area [4].

Nanosuspensions (NS) represent an innovative and versatile class of compounds that offer a sustainable solution for improving the delivery of drugs by increasing their solubility in water and lipids. The particle sizes of nanosuspensions are submicron (below 1 μm, generally between 200–500 nm), and they exhibit several unique physicochemical properties [5]. By definition, nanosuspensions are nanodispersed solid–liquid systems made of pure insoluble or partially soluble drugs in the solid state that are dispersed in an aqueous medium and stabilized by the use of a polymer or surfactant to prevent their sedimentation or aggregation.

For the first time in 1995, the first Gris-PEG nanosuspension drug was patented and marketed by Müller and his collaborators in the form of a nanosuspension as a sustained-release tablet, in which the active pharmaceutical ingredient is griseofulvin [6].

There are many technologies commonly utilized to prepare nanoparticles, such as supercritical fluid technology, emulsification solvent evaporation technique, emulsion diffusion method, melt emulsification method, lipid emulsion/microemulsion template, and nanojet technology. In addition to these manufacturing methods, there are also several methods developed by pharmaceutical product development laboratories, that have been classified into three categories: (i) top-down technologies (high-pressure homogenization [7], media milling); (ii) bottom-up technology (antisolvent precipitation method [8] and precipitation–ultrasonication, flash nanoprecipitation) and (iii) combination technology that starts first with a bottom-up process, followed by a top-down process [9]. Top-down methods involve breaking the drug into very small particles by grinding and/or homogenization methods, while bottom-up methods involve the use of the precipitation method to build the drug particles. Nevertheless, top-down methods have some disadvantages, such as long processing time, high energy input, metal contamination, and a wide particle size distribution compared to bottom-up methods [10].

Among the bottom-up methods, supercritical antisolvent precipitation, even though it requires low pressure and high temperature as a working procedure, can be widely used to obtain drugs with improved solubility rates, by adapting the working technique to atmospheric pressure and ambient temperature. Supersaturation is achieved by adding to the solution of a drug, insoluble or partially soluble, an antisolvent that has the role of starting the nucleation and solubilization processes of the drug. In the precipitation process of drug particles, it is useful to add stabilizers with the role of delaying or slowing down particle growth, and then a rapid drying process is required [10]. Among the improvements in the dissolution rate of a drug, the antisolvent precipitation methods lead to an enhancement in the compressibility and flowability parameters [11].

Wu C.Y., and Wang W. successfully prepared pure, excipient-free nanoparticles of curcumin, coumarin 6, nortriptyline hydrochloride, amitriptyline hydrochloride, and prochlorperazine dimaleate using the antisolvent precipitation method. They demonstrated that this method, by using five different preparation working protocols, can be further used to predict the size of these nanodrugs [12]. Li Z. et al. demonstrated the influence of three antisolvent precipitation parameters, namely, the feeding rate, the aging time, and the agitation speed, on the physical stability of amorphous solid drugs in the preparation process [13].

Rivaroxaban (RIV), 5-chloro-N-(((5S)-2-oxo-3-(4-(3-oxomorpholin-4-yl) phenyl)-1,3-oxazolidin-5-yl) methyl)-thiophene-2-carboxamide, is a direct oral active anticoagulant drug (factor Xa inhibitor) widely used to prevent and treat blood clots, reducing the risk of stroke in atrial fibrillation, deep vein thrombosis (DVT), and pulmonary embolism. RIV is a poorly water-soluble drug with a limited oral bioavailability, belonging to the Biopharmaceutical Classification System (BCS) class II. There are a few studies reported to enhance the solubility of RIV [14,15,16,17].

Hydroxypropyl methylcellulose (HPMC) is a semisynthetic, nonionic, hydrophilic polymer derived from cellulose with (1→4) linked β-d-glucose units and partially *O*-methylated or *O*-(2-hydroxypropylated) hydroxyl groups [18,19]. Its properties vary depending on the molar mass and degree of substitution, and solutions are stable at pH 3–11 and can undergo reversible gelation between 50–90 °C [20,21]. HPMC forms transparent and flexible films and is widely used due to its biocompatibility, biodegradability, and water solubility, both in medicine (controlled drug release) [22] and in industries such as food, cosmetics, textiles, paints, adhesives, and wastewater treatment [23,24].

In the large family of poloxamers, Poloxamer 188 (P188) is a non-ionic linear copolymer composed of one block of hydrophilic polyoxyethylene (38 moieties), neighboring one block of hydrophobic polyoxypropylene (29 moieties) [25]. Due to its biocompatibility and properties as a surfactant, it has been widely used in pharmaceutical, cosmetic, and industrial domains. Poloxamer-based nanoformulations have shown great potential in the pharmaceutical development for drug delivery, to improve drug solubilization and bioavailability [26,27].

Alamri AH et al. [15] developed rivaroxaban-loaded nanostructured lipid carriers with good dissolution behavior; however, the systems were not incorporated into a final pharmaceutical form and can be considered only a preliminary preformulation study. Meanwhile, Venkata Naga Jyothi Nakka and Srawan Kumar GY [28] studied the performance of rivaroxaban nanosuspension-based tablets. Their findings show excellent dissolution behavior, but the tablets contain sodium lauryl sulfate. Demir Huriye et al. [29] also demonstrated that rivaroxaban nanocrystals have better dissolution performance than rivaroxaban powder, but the obtained nanocrystals were not incorporated into a pharmaceutical dosage form. Similarly, Abd-Ali KJ and Toma NM [30] limited their study to the performance of the nanocrystals.

Our previously published studies describe the manufacturing and performance of solid dosage forms containing rivaroxaban. To enhance dissolution behavior, different methods were used: inclusion in various cyclodextrins and cocrystal formation with niacinamide. Incorporating rivaroxaban into various beta-cyclodextrin cavities produced tablets with improved dissolution compared to the original product (Xarelto). However, coating cellets with rivaroxaban–cyclodextrin dispersions appears to have low reproducibility [17], while incorporating lyophilized inclusion complexes into tablets is an expensive technique [16].

The cocrystallization approach [31] is a valid alternative, offering good reproducibility and low costs, but the results are not as favorable as those found for cyclodextrin inclusion complexes or the current nanosuspensions.

Overall, the goal and the novelty of this research is the use of the antisolvent precipitation method, as a bottom-up technology, for the preparation of three dry pharmaceutical nanosuspensions by varying the mass ratio between RIV (active pharmaceutical ingredient), Poloxamer 188 (nonionic surfactant as stabilizer), and HPMC (polymeric stabilizer) components as following 1:1:1, 1:2:1, and 1:1:2, respectively. This method allows the advancement of a practical solution for the development of new drugs with the active ingredient rivaroxaban with improved bioavailability. In the literature, it was widely used to prepare different formulations [32,33,34,35]. This research has been developed with the scope to enhance the water solubility of RIV and, subsequently, its dissolution characteristics. The influence of the concentration of P188 and HPMC and their interaction with RIV has been investigated using Fourier infrared spectroscopy, X-ray diffraction studies, and scanning electron microscopy. Also, their stability was investigated using the thermal analysis technique.

Generic production of rivaroxaban was permitted starting in January 2025. Consequently, rivaroxaban remains insufficiently studied regarding its incorporation into various pharmaceutical forms. Given rivaroxaban’s low solubility in water, it is essential to formulate it in suitable pharmaceutical systems that provide a high dissolution rate, which in turn ensures good absorption and bioavailability. The original Xarelto tablets contain sodium lauryl sulfate, which increases the dissolution rate of rivaroxaban. The main objective of our study was to develop rivaroxaban tablets that do not include sodium lauryl sulfate or other irritating or toxic excipients, as rivaroxaban is used for chronic treatment and, for some patients, lifelong administration may be necessary. The novelty lies in using both stabilizers (P188 and HPMC) for the preparation of rivaroxaban nanosuspensions. To our knowledge, all previously studied rivaroxaban nanosuspensions have included either SLS in the formulation or used other polymers for nanocrystal formation. The obtained nanosuspensions were used to formulate tablets, and the in vitro dissolution performance was assessed compared with a commercially available reference product, Xarelto^®^ 10 mg.

## 2. Materials and Methods

### 2.1. Materials

The micronized RIV (Form I) manufactured by Neuland Laboratories Limited was kindly donated by Labormed-Pharma SA, Romania. Poloxamer 188 (P188) (Poly(ethylene glycol)-block-poly(propylene glycol)-block-poly(ethylene glycol) copolymer) and Hydroxypropyl methylcellulose (HPMC) were provided by Sigma-Aldrich Chemie GmbH, Taufkirchen, Germany. Avicel^®^ PH 102, purchased from International Flavors and Fragrances Inc. IFF, New York, NY, USA, and Flowlac^®^ 100, was manufactured by Meggle GmbH & Co. KG (Wasserburg am Inn, Germany. EXPLOTAB^®^ was provided by JRS PHARMA GmbH & Co. KG, Rosenberg, Germany, and LIGAMED^®^ MF-2-V by Peter Graven (Venlo, NV, The Netherlands).

Acetonitrile (HiPerSolv CHROMANORM for LC–MS) was supplied by VWR (Leuven, Belgium). Formic acid (≥99.0%, Optima™ LC–MS grade) and sodium hydroxide (extra pure, 50 wt% aqueous solution) were obtained from Fisher Chemical (Thermo Fisher Scientific, Waltham, MA, USA). Ultrapure water (18.2 MΩ·cm at 25 °C) was produced using a Milli-Q EQ 7008 purification system (Merck Millipore, Burlington, MA, USA). The other reagents, including potassium phosphate monobasic, sodium acetate, acetic acid, and sodium dodecyl sulfate (SDS) were purchased from Merck KGaA (Darmstadt, Germany). All chemicals and solvents used were of analytical reagent grade. A Mettler Toledo AT261 balance with a sensitivity of 0.01 mg was used to weigh the materials.

### 2.2. Methods

#### 2.2.1. Preparation of Dry RIV Nanosuspensions by the Antisolvent Precipitation Method

Three dry pharmaceutical nanosuspensions were developed by varying the mass ratio between components (RIV, Poloxamer 188 and HPMC) to 1:1:1, 1:2:1 and 1:1:2.

The drug solution was prepared by dissolving 1 g of RIV powder in 10 mL of acetone. Meanwhile, Poloxamer 188 and HPMC were dispersed in 50 mL of distilled water, then slowly added to the RIV acetonic solution with continuous stirring at 800 rpm and room temperature using a Heidolph MR 3001K magnetic stirrer. After 90 min of mixing, the resulting suspensions were sonicated without heating at 37 kHz for 20 min in an Elmasonic S 40 (H) water bath, manufactured by Elma Schmidbauer GmbH, Singen, Germany. Finally, the samples were frozen and then freeze-dried for 10 h at −60 °C in a CoolSafe Basic and Pro lyophilizer produced by Labogene A/S, Allerod, Denmark.

#### 2.2.2. Preparation of RIV Physical Mixtures

The RIV, Poloxamer 188, and HPMC powders were mixed for five minutes at room temperature to create their physical mixtures.

#### 2.2.3. Physicochemical Characterization of the Dry RIV Nanosuspensions

A FTIR spectrophotometer (NICOLET 6700, Thermo Electron Corporation, Waltham, MA, USA) was used in transmission mode. The samples were mixed with KBr pellets (20 mg/cm^2^) (approximately 0.5% of the sample) using 1 mg of the sample with 200 mg of KBr, followed by compression under vacuum to ensure homogeneity. The prepared tablets were scanned within the 4000–400 cm^−1^ range, at a resolution of 4 cm^−1^.

The structures of samples were analyzed by room temperature X-ray diffraction measurements using a Bruker D8 Advance diffractometer at a Ni-filtered Cu-Kα radiation at a wavelength of 1.5418 Å. The X-ray tube functioned at 40 kV and 40 mA. The scan range was from 5 to 60° (2θ), with a step size of 0.02° and a counting time of 0.2 s per step. The degree of crystallinity was determined by profile fitting using a Pawley refinement procedure. The background was modeled using a flat background combined with polynomial terms (coefficients 1, 2, 3, and 1/x) to account for amorphous scattering and instrumental contributions. Diffraction peaks were described using a pseudo-Voigt shape function, while instrumental broadening was modeled using the Caglioti function. During refinement, the peak width parameters (U, V, W) and the peak shape parameter (Shape 1) were refined to optimize the agreement between the calculated and experimental patterns. The crystallinity (%) was calculated as the ratio of the integrated intensity of the crystalline diffraction peaks to the total scattered intensity (sum of crystalline and amorphous contributions) over the measured 2θ range.

Scanning electron microscopy (SEM) was accomplished using a Tescan Vega LMU Scanning Electron Microscope (Brno, Czech Republic) to characterize the morphologies of the samples. The apparatus functioned in low vacuum mode at 20 Pa, and the accelerating voltage was 10 kV.

The particle size distribution and zeta potential (ζ) were analyzed by dynamic light scattering (DLS) with a Malvern ZetaSizer Nano-ZS equipment (Malvern Instruments, Worcestershire, UK). Stock suspensions of RIV-P188-HPMC (1:1:1), RIV-P188-HPMC (1:2:1), and RIV-P188-HPMC (1:1:2) were prepared at a concentration of 1 mg mL^−1^ in water at pH 7. The samples were gently mixed in a tube roller for 1 h to ensure homogeneous dispersion. Subsequently, these stock suspensions were diluted to a final concentration of 50 µg mL^−1^ in water at pH 7. The diluted suspensions were sonicated for 10 min before the measurements were performed.

The thermal physical changes were investigated using a TG-DTA (NETZSCH STA 449 F3 Jupiter instrument, Selb, Germany). The samples were heated from room temperature to 600 °C, in alumina crucibles, at a heating rate of 10 °C/min. Nitrogen was used as a purge gas at a flow rate of 20 mL/min. The obtained experimental data were processed using NETZSCH Proteus—Thermal Analysis software, version 5.2.1.

#### 2.2.4. Formulation of Tablets Containing Dry RIV Nanosuspensions

Direct compression is the preferred manufacturing method for tablets and was also selected for the development of the fast-release tablets in this study due to its simplicity and the fact that the active ingredients are protected from heat and moisture. To guarantee that the finished tablets have suitable uniformity, pharmacotechnical and stability attributes, a directly compressed material with adequate flowability and compressibility is necessary [36]. Usually, lyophilized powders are difficult to incorporate into tablets by direct compression, as they have low flowability and compactability, being amorphous systems.

Therefore, one of the most important steps in the tablet development process is selecting the excipients, considering both their types and amounts.

Avicel^®^ PH 102 (microcrystalline cellulose) and Flowlac^®^ 100 (spray-dried lactose) were selected as fillers for their reliable filling, binding, and disintegrating qualities, and above all for their good flow and compressing properties, which are imperative for direct compression technology, particularly for amorphous ingredients that have poor flow ability [37].

Ligamed^®^ MF-2-V (magnesium stearate) was chosen for its gliding characteristics, and Explotab^®^ (sodium starch glycolate) was used as a superdisintegrant [38].

Tablets with a total weight of 200 mg and a concentration of 10 mg RIV were formulated by calculating the amounts of each component accordingly. Four series of tablets were manufactured: two contained the dry RIV nanosuspensions as active ingredients, and the other two contained the prepared physical mixtures. The formulations selected for each binary system are shown in Table 1.

#### 2.2.5. Tablets Manufacturing Process

The ingredients were individually sieved through a 20-mesh sieve and then weighed in the specified amounts. The active ingredients were mixed with the two fillers and the superdisintegrant in a CMP 12 Plexiglas cube mixer (Pharmag GmbH, Klipphausen, Germany) at 25 rpm for 15 min at room temperature (23 °C). Finally, magnesium stearate was added and blended for an additional two minutes, maintaining the same conditions. The materials were compressed at a compacting force of 10 kN using a single-post eccentric tablet press, Erweka EP-1 (Erweka, Germany), equipped with 8 mm flat punches. The machine was set to produce 200 mg tablets.

In the case of F5, a higher quantity of HPMC appears to result in a system with low flowability, which may be due to the high adhesive properties of the polymer. This creates strong bonds between the powder particles, reducing the plastic deformation of the materials [39,40,41]. However, F5 did not yield a suitable material, as it could not be directly compressed because the powder did not flow consistently from the funnel to the die. The powder adhered to the funnel and created multiple voids during the compression process, so F5 and F6 were eliminated from further analysis.

### 2.3. Tablets Characterization

#### 2.3.1. Organoleptic Properties

The European Pharmacopoeia guidelines were used to assess the tablets’ appearance [42].

#### 2.3.2. Dimensions (Diameter and Thickness)

A VK 200 tablet hardness tester from Vanderkamp, USA, was used to measure the thickness and diameter of ten tablets from each batch.

#### 2.3.3. Mass Uniformity

Twenty tablets of each formulation were weighed individually, and the average mass was calculated [42].

#### 2.3.4. Hardness

The hardness was measured using the VK 200 tablet hardness tester. It is defined as the force required to crush tablets positioned between the two anvils of the device. Ten tablets from each batch were tested.

#### 2.3.5. Friability

The Vankel friabilator was used to test ten tablets from each batch. After weighing, the tablets were placed in the device’s drums and rotated for five minutes at 30 rpm. To determine mass loss during rotation, the tablets were dedusted and weighed again. According to compendial standards, the maximum acceptable loss is 1.0% [42].

#### 2.3.6. In Vitro Disintegration Time

In distilled water media heated to 37 ± 0.5 °C, six tablets from each formulation were evaluated for disintegration behavior in accordance with European Pharmacopoeia requirements [42]. An Erweka DT 3 device, manufactured by Erweka GmbH in Germany, was used to measure the time in seconds required for complete disintegration.

### 2.4. In Vitro Dissolution Study

In vitro dissolution of rivaroxaban from the experimental tablets were evaluated using a Vision G2 Classic 6 Dissolution Tester (Teledyne Hanson, Chatsworth, CA, USA), configured with USP Apparatus II (paddles). Dissolutions were conducted at 37.0 ± 0.5 °C and 75 rpm, in line with the USP guidelines for rivaroxaban tablets [43]. Each vessel contained 900 mL of dissolution medium, with testing performed in two media: a 0.022 M sodium acetate buffer at pH 4.5 containing 0.2% SDS (the compendial medium for 10 mg rivaroxaban tablets) and a 0.05 M phosphate buffer at pH 6.8 without surfactants.

Aliquots of 1.5 ± 0.1 mL were withdrawn at 5, 10, 15, 20, 30, 45, 60, 90, 120, and 180 min. After each withdrawal, an equivalent volume of fresh, preheated medium was added to maintain sink conditions. The collected samples were filtered through a 0.45 μm polyethersulfone membrane prior to analysis. All dissolution runs were performed in triplicate.

For a comprehensive assessment of the drug release performance, the same dissolution tests were also performed on a commercially available reference product, Xarelto^®^ 10 mg (Bayer AG, Leverkusen, Germany), and on pure micronized rivaroxaban (Form I), allowing direct comparison of the nanosuspension-based formulations with both the commercial benchmark and the unformulated drug substance.

Rivaroxaban quantification was performed using a reversed-phase HPLC method adapted from a previously reported procedure [17]. Analyses were carried out on a Jasco 4000 Series HPLC system (JASCO Corporation, Tokyo, Japan) equipped with a Kinetex^®^ C18 column (100 × 3 mm, 2.6 μm, Phenomenex, Torrance, CA, USA), maintained at 45 °C. The separation was achieved under isocratic conditions using a mobile phase consisting of 0.1% (*v*/*v*) aqueous formic acid and acetonitrile, mixed at an optimized ratio of 62:38 (*v*/*v*). Detection was performed at 250 nm. Calibration curves were constructed using standard solutions of rivaroxaban in the concentration range of 0.156–20 μg/mL, and were used to quantify the dissolved drug in the collected samples.

### 2.5. Data Analysis

#### 2.5.1. Dissolution Efficiency

Dissolution efficiency (DE%) was calculated for each formulation at each dissolution medium using the trapezoidal rule to compute the area under the dissolution–time curve (AUC) [44], according to the equation:

(1) $$DE\left(\%\right)=\frac{{AUC}_{0\to t}}{100\cdot t}\times 100$$

where ${AUC}_{0\to t}$ is the area under the dissolution–time curve from t = 0 to the final time point t (120 min for pH 4.5 medium; 180 min for pH 6.8 medium), calculated by trapezoidal integration using mean percent dissolved values at each time point. Values of cumulative dissolution exceeding 100% (arising from measurement uncertainty) were capped at 100% prior to integration.

#### 2.5.2. Similarity Factor (f_2_)

Profile similarity was evaluated using the similarity factor f_2_, calculated according to the model-independent approach recommended in regulatory guidelines [45,46,47]:

(2) $$f_{2}=50\cdot log\left\{{\left[1+\sum_{t=1}^{n}{\left(R_{t}-T_{t}\right)}^{2}\right]}^{-0.5}\cdot 100\right\}$$

where *n* is the number of sampling time points, $R_{t}$ is the mean cumulative percent dissolved of the reference product (Xarelto^®^ 10 mg), and $T_{t}$ is the mean cumulative percent dissolved of the test formulation at time point t. An f_2_ value ≥ 50 indicates similarity between the two dissolution profiles. In accordance with FDA and EMA guidance [45,47], f_2_ was not calculated when more than one time point of either the reference or test profile exceeded 85% dissolution, as this condition renders the metric uninformative. For the pH 4.5 medium, where Xarelto^®^ 10 mg and F3 both exceeded 85% before t = 15 min, DE–based comparison was used as the primary metric instead.

#### 2.5.3. Dissolution Kinetic Modeling

Cumulative dissolution data obtained in pH 6.8 phosphate buffer were fitted to zero-order, first-order, Higuchi, and Korsmeyer–Peppas (KP) models in order to characterize the drug release mechanism [48,49]. In all cases, model fitting was performed using non-linear least squares. The KP model was applied only to the initial portion of the dissolution profiles (Q ≤ 60%), in accordance with its theoretical validity range [50,51]. For formulations that did not exceed 60% dissolution throughout the experiment (F2, F4, RIV, and Xarelto^®^ 10 mg), the full dataset was considered inherently within the applicable range.

Model selection was based on the coefficient of determination (R^2^). The release exponent (*n*) obtained from the KP model was used to interpret the release mechanism, distinguishing between Fickian diffusion (*n* ≤ 0.45), anomalous transport (0.45 < *n* ≤ 0.89), and Case II transport (*n* > 0.89) [50].

### 2.6. Statistical Analysis

All dissolution data are presented as mean ± standard deviation (SD). Differences among formulations at selected time points were evaluated using one-way analysis of variance (ANOVA). When statistically significant, post hoc pairwise comparisons were performed using the Bonferroni correction to control the family-wise error rate.

Pairwise comparisons between each formulation and the commercial reference product (Xarelto^®^ 10 mg) were performed using independent two-sample *t*-tests. Statistical significance was considered at *p* < 0.05 unless otherwise specified.

All calculations, including trapezoidal integration, curve fitting, and statistical analyses, were performed using GraphPad Prism Software version 10.0.0 (GraphPad, La Jolla, CA, USA).

## 3. Results and Discussion

### 3.1. Physicochemical Characterization of the Dry RIV Nanosuspensions

#### 3.1.1. FTIR Characterization of Materials

**Figure 1:**
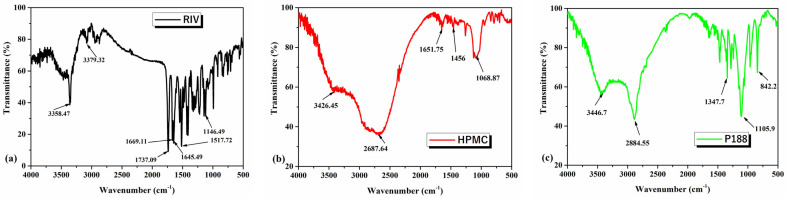

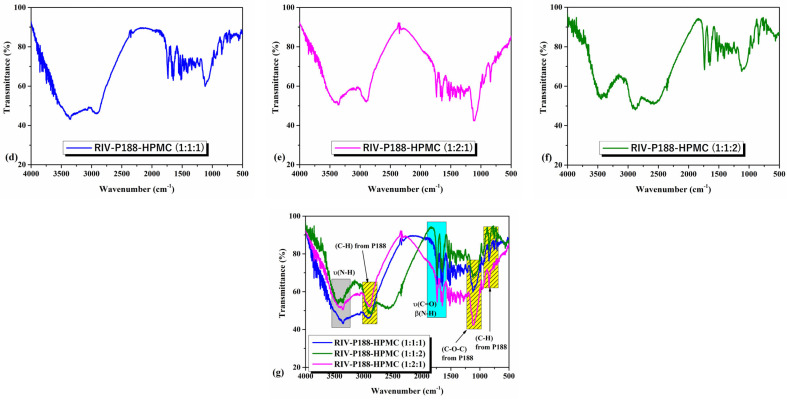
FTIR spectra of (**a**) rivaroxaban (RIV), (**b**) HPMC, (**c**) P188, (**d**) RIV-P188-HPMC (1:1:1), (**e**) RIV-P188-HPMC (1:2:1), and (**f**) RIV-P188-HPMC (1:1:2), and (**g**) combined FTIR spectra of the three dry nanosuspensions.

The FTIR spectra of raw materials and their nanosuspensions in different molar ratios are shown in Figure 1. From FTIR data, information on changes in bond and functional group peaks leads to a useful basis for establishing the type of interaction between compounds.

The main characteristic absorption bands from the FTIR spectrum of RIV (Figure 1a) are: at 3358.47 cm^−1^ is assigned to secondary amide υ(N-H), at 1669.11 cm^−1^ attributed to υ(C=O), and at 1645.49 cm^−1^ assigned to stretching frequency β(N-H). The stretching vibration of carbonyl C=O appears at 1737.09 cm^−1^, and the stretching vibration of C-C from aromatic ring appears at 1517.72 cm^−1^. The characteristic band of Rivaroxaban form I appears at 1146.49 cm^−1^ [52,53].

The FTIR spectrum of HPMC (Figure 1b) shows the following adsorption bands: at 3426.45 cm^−1^ assigned to hydroxyl (OH) stretching frequency and at 1385.14 cm^−1^ to the bending vibration of the OH group. The band at 1068.87 cm^−1^ is attributed to the stretching vibration of the C-O group [54]. The shoulder observed between 2800–3000 cm^−1^ is attributed to ν(C–H) from the basic skeleton of the saccharide unit [55].

The FTIR spectrum of P188 (Figure 1c) shows the main characteristic peaks, as follows: at 2884.55 cm^−1^ assigned to aliphatic C-H stretching vibration, at 3446.7 cm^−1^ assigned to O-H stretching vibration, at 1347.7 cm^−1^ assigned to O-H deformation vibrations (in-plane bend), at 1105.9 cm^−1^ assigned to aliphatic C-O-C stretching vibration, and at 842.2 cm^−1^ assigned to C-H deformation vibration. The FTIR spectrum is characteristic of a polyoxyethylene–polyoxypropylene block copolymer.

The FTIR spectra of the three dry nanosuspension formulations were compared with those of the individual raw materials (Figure 1d–f). The main characteristic bands of rivaroxaban and P188 were also identified in the formulations, without the appearance of significant new bands, indicating the absence of chemical reactions between the drug and the excipients (Figure 1g). However, slight shifts and changes in the intensity of some bands were observed, which are attributed to physical interactions (Table 2) [56].

The results from Table 2 showed shifts between 0.5 and 6 cm^−1^, which are minor, suggesting a weak interaction strength associated with physical intermolecular interactions without evidence of new chemical bond formation. As it was shown in Figure 1g, the FTIR profile of RIV-P188-HPMC (1:1:1) and RIV-P188-HPMC (1:2:1) is quite similar, while the FTIR spectrum of RIV-P188-HPMC (1:1:2), with a higher concentration of HPMC, shows a broadening of the FTIR peak between 3000 and 2500 cm^−1^, due to a higher amount of HPMC. These FTIR results confirm the compatibility of all the components and the maintenance of the chemical structure of the drug in the formulation without any significant interaction among these ingredients [57].

#### 3.1.2. XRD Analysis

**Figure 2:**
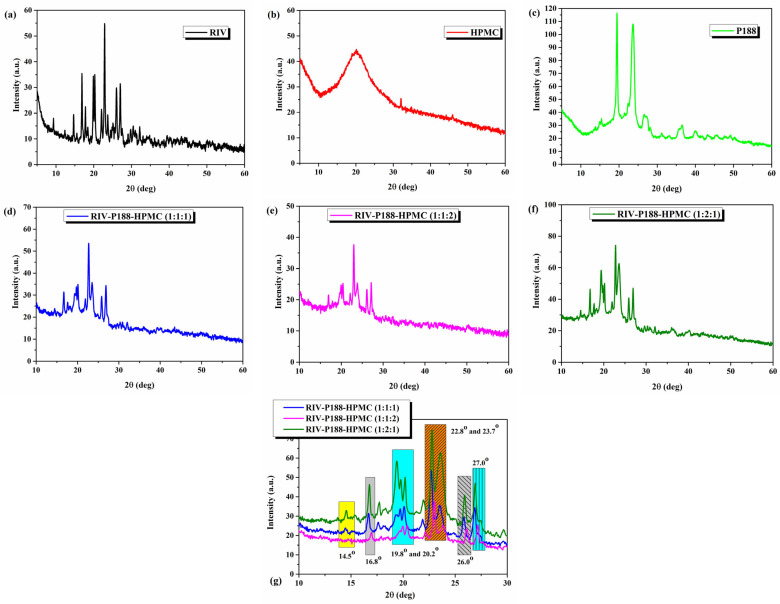
X-ray diffractograms of (**a**) rivaroxaban (RIV), (**b**) HPMC, (**c**) P188, (**d**) RIV-P188-HPMC (1:1:1), (**e**) RIV-P188-HPMC (1:1:2), (**f**) RIV-P188-HPMC (1:2:1), and (**g**) the three nanosuspension superposed.

The X-ray diffraction patterns of the studied materials are shown in Figure 2.

The XRD spectrum of RIV (Figure 2a) reveals intense diffraction peaks at 2θ = 14.6°, 16.8°, 19.9°, 20.3°, 22.8°, 26.0° and 27.0°, proving its crystalline character [14]. The calculated degree of crystallinity for RIV is 51.35%. The X-ray diffraction pattern of HPMC (Figure 2b) shows one broad diffused peak at approximately 2θ = 20° suggesting the amorphous nature of HPMC [58]. The crystallinity is 39.9%.

The XRD results of P188 are shown in Figure 2c. Two diffraction peaks were observed at 2θ = 19.4° and 23.7°, respectively [59], confirming its crystalline polymeric nature (degree of crystallinity of 71.67%).

The XRD pattern of the three formulations (Figure 2d–g) showed a significant reduction in the intensity of RIV peaks or their displacement in the dry nanosuspensions, indicating a decrease in the crystallinity degree and a possible partial amorphization of the drug as a result of the rapid precipitation process by the antisolvent method (bottom-up). The degree of crystallinity was 31.72% for RIV-P188-HPMC (1:1:1), 35.29% for RIV-P188-HPMC (1:2:1), and 17.88% for RIV-P188-HPMC (1:1:2). Also, the two XRD peaks of P188 are slightly displaced, and their intensity is reduced. The greatest reduction in the RIV peaks is shown for RIV-P188-HPMC (1:1:2), which contains a higher concentration of HPMC. The absence of the highly crystalline structure suggests that rivaroxaban is dispersed molecularly or in the form of very fine particles in the polymeric matrix formed by HPMC and Poloxamer 188, thus supporting the formation of the nanosuspension system through the antisolvent precipitation technique.

The reduction in the intensity of the XRD peaks of Rivaroxaban in nanosuspensions indicates a decrease in the degree of crystallinity and a possible partial amorphization of the drug, as a result of the rapid precipitation process by the antisolvent method (as an effective bottom-up technique). Also, the decrease in the peaks corresponding to Poloxamer 188 suggests its involvement in the stabilization of the system and uniform dispersion of the particles. This reduction in crystallinity is directly correlated with the improvement of the dissolution profile, since the amorphous form presents a higher free energy and an apparently increased solubility compared to the crystalline form. In addition, the reduction in particle size and improved dispersion of RIV are attributed to the presence of P188, which increases the specific surface area, enhances steric hindrance, improves dispersion, and promotes wetting, thereby facilitating rapid drug dissolution [60]. The presence of HPMC, a viscous polymer, has also been successfully employed as a stabilizer for RIV. The same effect was observed in the stabilization of itraconazole nanocrystals by Mou D. et al. [61]. Regarding the differences observed in the nanosuspensions with different concentrations of HPMC demonstrate that the concentration of HPMC is an important factor in the nanosuspension preparation. A high concentration of HPMC contributes to lowering its efficacy due to the decrease in surface coverage capacity to form a steric hindrance, and even to aggregation [60,62]. Thus, the XRD results support the experimental observations regarding the formation of nanosuspensions, especially in the case of formulations with a high Poloxamer 188 contents, where the stabilization effect is more pronounced.

#### 3.1.3. SEM Analysis

**Figure 3:**
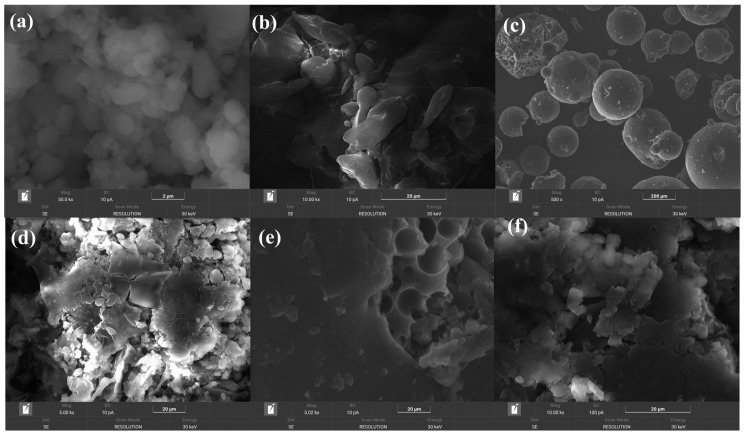
SEM images of (**a**) rivaroxaban (RIV), (**b**) HPMC, (**c**) P188, (**d**) RIV-P188-HPMC (1:1:1), (**e**) RIV-P188-HPMC (1:1:2), and (**f**) RIV-P188-HPMC (1:2:1).

Scanning electron microscopy is useful to elucidate the morphology of the nanosuspensions. SEM images of RIV, HPMC, P188, and the three nanosuspensions are shown in Figure 3.

SEM microstructure of RIV (Figure 3a) presents well-defined crystalline particles with regular shapes and a relatively smooth surface, indicating its crystalline nature. HPMC appears as an amorphous mass, with a lamellar structure, without distinct crystalline forms (Figure 3b). SEM image revealed that Poloxamer 188 (Figure 3c) presents predominantly spherical crystalline particles as spheres, with a smooth surface and uniform morphology, suggesting a compact structure and good dispersion capacity [63].

SEM images of nanosuspensions formulated with rivaroxaban, Poloxamer 188, and HPMC revealed a morphology dependent on the molar ratio of the components. In the RIV-P188-HPMC (1:1:1) dry nanosuspension (Figure 3d), the particles were relatively uniform, with a slightly irregular surface, and the P188 was observed as spherical particles dispersed in the amorphous HPMC matrix. In the case of the nanosuspension with a double ratio of HPMC (RIV-P188-HPMC (1:1:2) (Figure 3e), the HPMC polymer matrix was denser, giving a slightly irregular surface and a slight tendency to agglomerate, although the P188 particles remained spherical. Increasing the proportion of P188, compared with RIV and HPMC, in the RIV-P188-HPMC (1:2:1) (Figure 3f) led to more spherical particles with a smooth surface, the nanosuspension becoming more homogeneous and stable, suggesting a uniform dispersion of rivaroxaban.

In all nanosuspension formulations, rivaroxaban crystals were no longer so clearly visible, indicating the transition of the drug to a less crystalline and partially amorphous state by reducing the particle size. The literature data showed that the enhanced solubility and bioavailability of drugs, as demonstrated by the Ostwald–Freundlich equation, are due to an increase in surface area as a result of a reduction in particle size [64]. The SEM results also suggest that the molar ratio of excipients influences the morphology and dispersion of RIV in the nanosuspension, as demonstrated by XRD analysis.

#### 3.1.4. DLS Analysis

**Figure 4:**
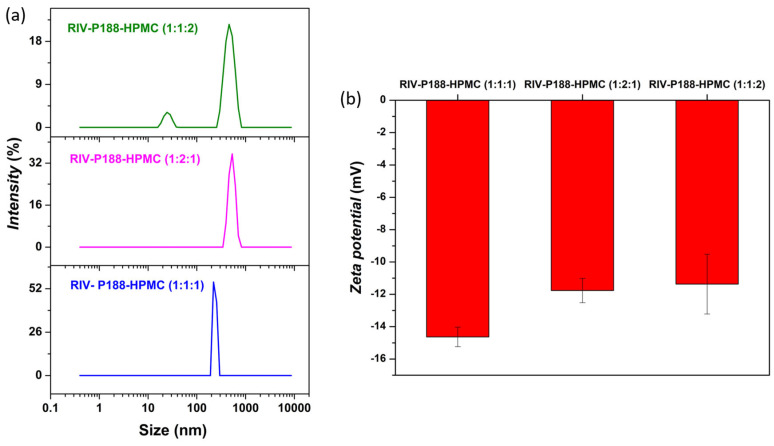
(**a**) Particle size distribution, and (**b**) zeta potential for the RIV-P188-HPMC systems.

To gain deeper insight into particle size and colloidal stability in suspension, DLS analysis was carried out. The distribution of particle size for the three samples of the RIV-P188-HPMC system is represented in Figure 4a.

The RIV-P188-HPMC (1:1:1) showed a narrow distribution size at 342.7 ± 70.60 nm, indicating the formation of relatively uniform colloidal structures stabilized by the polymer matrix. In contrast, the P188-rich system (1:2:1) showed a slightly broader size distribution at 564.37 ± 60.80 nm, consistent with the formation of larger, more hydrated assemblies. The HPMC-rich system (1:1:2) exhibits a bimodal distribution, consisting in a minor population at 21.14 ± 2.65 nm (≈10%) and a dominant population of 389.67 ± 62.62 nm (≈90%), indicative of a heterogeneous sample. The DLS measurements performed on the as-prepared RIV-P188-HPMC dispersions revealed a highly polydisperse system (PDI between 0.756 and 1), indicating the presence of larger aggregates. The obtained results correlate well with previously reported data on Rivaroxaban-based polymeric systems [65].

Furthermore, to gain insight into the surface charge characteristics and colloidal stability of the dispersed systems, the zeta potential was determined. Generally, ζ-potential values exceeding ±30 mV are typically associated with electrostatically stable dispersions, while smaller values indicate an increased tendency toward aggregation. However, for the RIV–P188–HPMC systems, steric stabilization is expected to play an important role, due to the presence of P188 and HPMC, and the obtaining of smaller ζ-potential than ±30 mV values is not considered poor colloidal stability [65].

Figure 4b presents the zeta potential values of RIV-P188-HPMC systems measured in water at pH 7. The ζ-potential for the RIV-P188-HPMC systems was −14.6 ± 0.6 mV, −11.8 ± 0.7 mV, and −11.4 ± 1.8 mV for the 1:1:1, 1:2:1, and 1:1:2 formulation/ratios, respectively. The increase in P188 or HPMC content slightly reduced the magnitude of the ζ-potential values. Altogether, all the relatively low ζ-potential values indicated a weak electrostatic stabilization, but combined with steric stabilization induced by hydrophilic HPMC polymer and P188 copolymer, proved to be suitable to maintain colloidal stability of RIV-P188-HPMC dispersions. The zeta-potential measurements were conducted on two consecutive days using aliquots from the stock dispersions. The ζ-potential values obtained remained consistent within the experimental error range.

#### 3.1.5. Thermal Analysis

**Figure 5:**
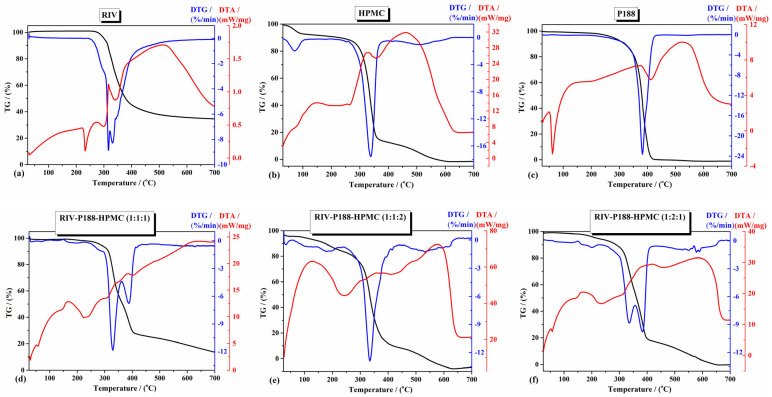
Thermal curves (TG-DTG-DTA) of (**a**) rivaroxaban (RIV), (**b**) HPMC, (**c**) P188, (**d**) RIV-P188-HPMC (1:1:1), (**e**) RIV-P188-HPMC (1:1:2), and (**f**) RIV-P188-HPMC (1:2:1).

The TG-DTA technique is a useful technique in which the registered thermal effects, like melting, any phase transition, and decomposition processes, provide the thermal stability of the studied materials. The TG-DTA curves of the studied raw materials and their dry nanosuspensions are shown in Figure 5. The TGA curve of RIV shows no mass loss before its melting at *T*_min_ = 231.9 °C (on DTA curve, red line from Figure 5a). The sharp endothermal peak, which appears on the DTA curve assigned to the melting process, indicates that the RIV compound is in a crystalline form, as suggested by its XRD pattern. The absence of mass loss and any thermal effect before melting demonstrates that the compound is anhydrous and contains no surface moisture or crystallization water. RIV starts to decompose at about 270 °C (onset temperature on the TG curve). At 700 °C, around 34.5% residue was found.

TGA of HPMC (Figure 5b) shows a multi-stage decomposition process in three steps (i) the first step between 25–114 °C represent the loss of moisture content with *T*_min_ = 69 °C on DTG curve and *T*_DTA_ = 76.7 °C, a mass loss of about 7.43%; (ii) the second step is due to a decomposition process between 190 and 365 °C (*T*_min_ = 336 °C on DTG curve and *T*_max_ = 324 °C on DTA curve, a mass loss of about 78%); (iii) the third step is due to a slight decomposition, occurring between 365 and 650 °C (*T*_min_ = 509 °C on DTG curve and *T*_max_ = 461 °C on DTA curve, a mass loss of about 14.57%) [66].

TGA curve for P188 (Figure 5c) exhibits a sharp endothermic peak at 62 °C, corresponding to the melting process. The sample remains stable until around 200 °C without any mass loss. The decomposition starts at about 240 °C until 700 °C. No residue was found after the decomposition process [67].

Thermogravimetric analysis (TGA) revealed differences between the degradation profiles of the raw materials and those of the three formulations (Figure 5d–f). In the case of the formulations, shifts in degradation temperatures and changes in mass loss steps were observed, suggesting an improved stabilization profile of dry nanosuspensions (Table 3). The thermal stability of the nanosuspensions increases in the following order: RIV-P188-HPMC (1:1:1) > RIV-P188-HPMC (1:2:1) > RIV-P188-HPMC (1:1:2).

In the formulated systems, the thermal behavior reflects the combined contribution of the components, indicating no new degradation pathways (as was shown in Figure 5 and the major weight loss from Table 3). The presence of polymeric excipients shows an important contribution in improving the thermal stability of the drug by forming a stabilizing matrix.

Another factor is the change in the melting point value of the drug due to a decrease in its crystallinity [68]. It was observed from Table 3 that for RIV-P188-HPMC (1:1:1) and RIV-P188-HPMC (1:2:1) nanosuspensions, there are some changes in the melting points of RIV and P188, and in the RIV-P188-HPMC (1:1:2) sample, they disappeared.

The presence of HPMC and Poloxamer 188 contributed to an improved thermal stability, indicating the formation of a more stable system. The literature shows that the stability of nanosuspensions is due to the combination of nonionic surfactants and polymers, through steric mechanisms. These depend on the weight and length of the used steric stabilizer [69]. Based on the data from Table 3, it can be observed that the higher HPMC concentration leads to a decrease in the decomposition temperature. Xia D. et al. demonstrate that the HPMC contributes to an enhanced viscosity and slows down the particle growth by inhibiting the diffusion of the molecules of solute [70].

In conclusion, FTIR, XRD, SEM, and TGA results indicate that rivaroxaban is compatible with the used excipients (P188 and HPMC). RIV is well dispersed in the matrix and has a reduced particle size, without major drug—polymer interactions, but with possible physical ones, contributing to the overall stability of the system.

### 3.2. Tablets Characterization

**Figure 6:**
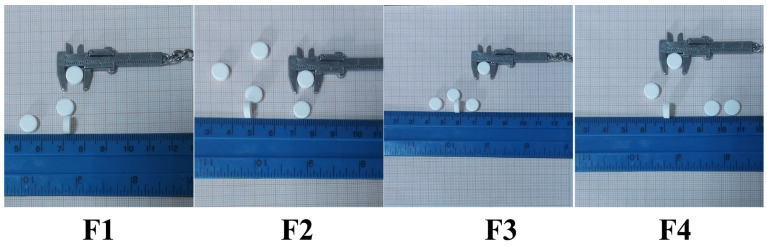
Tablets appearance.

The resulting tablets are white, round in shape, and have smooth and uniform surfaces (Figure 6).

The tablets’ mass and dimensions (thickness and diameter) varied only slightly, suggesting that the materials were sufficiently flowable to fill the dies uniformly. Small differences both within and between the tablet series show that the excipients, rather than the type of active ingredient, have an impact on the mass and size of the tablets. Each of the four formulations produced tablets with a diameter of 8 mm, a thickness of 3.00–3.03 mm, and a weight of about 200 mg. These results demonstrate that the excipients and compression conditions were appropriately selected and comply with the requirements of the European Pharmacopoeia [42]. Achieving uniform mass and size ensures the tablets’ consistent and appropriate dosage.

Jakubowska E and Ciepluch N. described two processing sources of failed mass uniformity: segregation of initially well-mixed material during handling or compression, and poor mixing of ingredients resulting in failure to achieve the required blend uniformity as an intermediate. Their research focused on demixing and the resulting uneven distribution of materials during various stages of tablet production, demonstrating the importance of reducing powder segregation to ensure uniform tablets [71].

Yohana Chaerunisaa A. et al. [72] stated that among all direct compression fillers, MCC is the most compressible and have the most capacity and propensity for dilution. Even at moderate compression forces, a powerful compact is created by the plastic deformation of MCC particles under compaction forces, which results in an incredibly large number of clean surfaces brought into contact. Saigal N et al. [73] described MCC as a self-disintegrating binder with minimal lubrication demands as to its dry binding capabilities, due to its remarkably low coefficient of friction and residual die wall pressure.

Lamešić D et al. [74] reported that spray-dried lactose has a high specific surface area and intra-particle porosity, resulting in improved uniformity of tablet content. Also, Rojas J. et al. [75] proved that the isodiametric form of spray-dried particles facilitates particle rearrangement in the die during tableting, improves compaction behavior, and leads to tablets with uniform mass.

Given these considerations, the consistency of tablet mass and dimensions demonstrates proper filler selection in each of the four studied formulations.

The hardness, on the other hand, differs significantly between batches, indicating variable compactability of the blends at the same compression force. Hardness ranges from 59 ± 1.52 N (F4) to 71 ± 1.75 N (F1), with intermediate values of 67 ± 1.37 N (F2) and 62 ± 1.06 N (F3). The nanosuspension-based tablets show higher mechanical resistance than the physical mixture-based tablets, probably due to the moisture contained in the lyophilized systems. The residual water acts as a binder in the tablets, leading to particles interlocking. Since the excipients are the same, only their amounts and the method used to create the nanosystems significantly affect the blends’ plasticity and elasticity. It is clear that tablets containing physical mixtures have lower mechanical resistance than those incorporating nanosuspensions. Additionally, F1 and F2, which contain higher amounts of directly compressible fillers, have greater hardness than the corresponding F3 and F4 batches. The mechanical resistance of all tablets is acceptable in terms of both hardness and friability, despite notable variations among the formulations. Although all values fall within the compendial limit (<1.0%), the tablets’ friability is more consistent between batches. F3 had the highest value, with a weight loss of 0.09%, while F4 registered a friability of 0.06%, and F1 and F2 did not suffer any loss during testing.

Diametrical compression force data are recorded using the diametral compression test, also known as the resistance to fracture test or tablet breaking force test. The mechanical strength of tablets depends on the ductile or brittle characteristics of the compression blend [76]. According to Adeleye OA. the impact of compression force on tablets is assisted by factors such as the material properties of the drug and excipients, formulation, and processing, all of which affect mechanical strength and release characteristics [77].

It is well known that lubricants reduce the mechanical strength of tablets by acting as a physical barrier that prevents particle–particle interactions, which affect the formation of inter-particulate bonds. For this reason, only magnesium stearate was chosen as the lubricant in the present study.

Sun CC and Hao H. [78] demonstrated that improved compaction qualities result from the slip planes in the crystal structure, thereby increasing tablet strength.

According to Osei-Yeboah F. and Sun CC. [79], tablet friability is influenced by various characteristics, including tablet size, shape, surface roughness, and tablet tensile strength or breaking force. Because friability directly evaluates tablet performance, the tablet friability profile (friability as a function of compaction force or pressure) can more accurately assess a formulation’s manufacturability than the compressibility profile.

The way tablets disintegrate significantly affects drug release behavior. In immediate-release formulations, the disintegration process is especially important. The studied tablets showed short disintegration times: 106 ± 3 s for F1, 115 ± 4 s for F2, 93 ± 1 s for F3, and 104 ± 3 s for F4, demonstrating that the included nanosuspensions result in faster disintegration than the simple physical mixtures. The primary mechanism by which sodium starch glycolate functions as a disintegrant is swelling; when its particles come into contact with water, their volume expands in all directions, enhancing liquid penetration [80].

The clear advantage is demonstrated by the direct compressible excipients, as they are more responsible for the disintegration behavior of the tablets. It is also evident that a higher amount of nanosuspension in the tablet formulation leads to a shorter disintegration time. This may be due to the higher concentration of Poloxamer 188, a surfactant that enhances disintegration by improving the wetting of solid systems [81], or because the nanosuspension allows for faster water penetration into the tablet. In the solidified material, lyophilization often results in a very porous and fragile structure that promotes rapid water wicking and tablet breakage [82].

The concentration of the superdisintegrant was appropriately selected, as shown by the short disintegration times of all tablet batches. Additionally, the ratio between the components of the tertiary system significantly affects disintegration performance, and the 1:2:1 ratio (RIV-P188-HPMC) appears to lead to faster disintegration.

Spray-dried lactose and microcrystalline cellulose (MCC) work together to enable quick tablet disintegration. Spray-dried lactose functions as a water-soluble binder, forming porous networks that allow quick water penetration and subsequent disintegration, particularly at higher concentrations, while MCC acts as a swelling agent, drawing water into the tablet [83].

The study by Kondo A et al. [84] demonstrated that the total amount of disintegrant in the tablet is more important than the variation in its distribution. Also, Beverly Nickerson et al. [85] established a linear relationship between the tablets’ disintegration and dissolution behaviors.

Markl D and Zeitler JA [86] stated that reproducible and complete disintegration of the tablet upon exposure to the dissolution medium is crucial for achieving dependable clinical performance of the dosage form, since without disintegration, only the active ingredient near the tablet’s surface would be able to dissolve. For immediate-release tablets, this typically delays the onset of dissolution and reduces the drug’s bioavailability. To facilitate quicker release of drug particles from the tablet matrix and increase the surface area for subsequent dissolution, disintegration agents are added to the formulation to promote the breakup of the tablets into their constituent particles.

Considering the study results and their correlation with the previously mentioned observations, it can be seen that an accurate selection of excipients for the proposed RIV-nanosuspension tablet formulations was achieved.

### 3.3. In Vitro Dissolution Profiles

**Figure 7:**
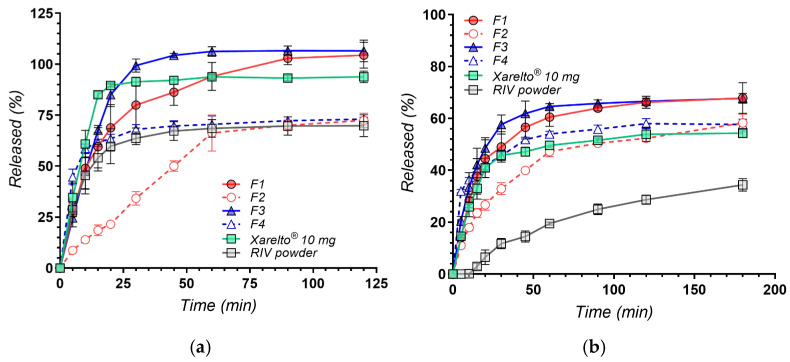
In vitro dissolution profiles of RIV from nanosuspension-based formulations (F1 and F3) and physical mixtures (F2 and F4), compared to pure micronized RIV and the commercial reference Xarelto^®^ 10 mg, in two different media: (**a**) pH 4.5 sodium acetate buffer with 0.2% SDS and (**b**) pH 6.8 phosphate buffer. Each point represents mean ± SD (*n* = 3).

The dissolution profiles of RIV from the investigated formulations in pH 4.5 acetate buffer containing 0.2% SDS and in pH 6.8 phosphate buffer are presented in Figure 7.

The use of these two media enables evaluation of formulation performance under both solubilizing (sink-like) and solubility-limited (non-sink) conditions [87]. The slightly acidic medium containing SDS corresponds to compendial testing conditions and provides a high solubilization capacity through micellar incorporation, thereby minimizing the impact of intrinsic drug solubility and emphasizing differences in dissolution kinetics. In contrast, the phosphate buffer at pH 6.8, in the absence of surfactant, imposes a thermodynamic constraint on dissolution and is more representative of intestinal conditions where solubility limitations govern drug release.

This dual approach is particularly relevant for RIV as a Biopharmaceutics Classification System class II compound, for which oral absorption is primarily dissolution rate-limited rather than permeability-limited [88]. Consequently, evaluating dissolution behavior under both permissive and restrictive conditions provides complementary insight into formulation performance, allowing differentiation between systems that enhance dissolution kinetics and those capable of increasing the extent of drug release under physiologically relevant constraints [89].

In the surfactant-containing medium (pH 4.5 with SDS), all formulations exhibited rapid drug release, with clear quantitative differences in both rate and extent. The nanosuspension-based formulation F3 showed the fastest release, reaching 85.0 ± 5.8% at 20 min and achieving near-complete dissolution by 30 min (99.4 ± 3.2%). F1 displayed a slightly slower but still rapid profile, while the commercial reference (Xarelto^®^ 10 mg) exhibited comparable early-time release (85.1 ± 1.6% at 15 min) and reached a plateau at later time points.

In contrast, the physical mixtures (F2 and F4) and the micronized drug showed substantially lower dissolution, plateauing at approximately 70% after 120 min. Dissolution efficiency (DE%) confirmed this ranking, with F3 (90.1%) and Xarelto^®^ (86.8%) showing the highest values, followed by F1 (83.9%), while F4 (67.1%), micronized RIV (63.1%), and F2 (51.9%) were markedly lower.

A quantitative summary of dissolution performance across both media, including representative time points and dissolution efficiency (DE%), is presented in Table 4.

Statistical analysis (one-way ANOVA, t = 120 min) indicated significant differences among formulations (*p* < 0.001). Post hoc Bonferroni comparisons showed that F1 and F3 did not differ significantly from each other (*p* = 0.374), but both exhibited significantly higher dissolution than the physical mixtures (*p* < 0.001).

As both F3 and the reference product exceeded 85% dissolution before 15 min, the similarity factor (f_2_) was not applicable under these conditions according to regulatory guidance [47]. Consequently, DE% was considered the most appropriate comparative metric. At later time points, the dissolution profiles of the rapidly releasing formulations became comparable, reflecting the limited discriminative capacity of the medium under sink conditions.

The quantitatively superior early dissolution of F1 and F3 relative to F2, F4, and the micronized API can be attributed to size-dependent and interfacial effects characteristic of nanosuspension systems. Particle size reduction increases the effective surface area available for dissolution, as reflected by the higher DE% values and faster initial release observed for these formulations, consistent with the Noyes–Whitney relationship.

In parallel, the apparent saturation solubility of nanosized particles has been reported to increase with decreasing particle size, as described by the Ostwald–Freundlich relationship, commonly attributed to Kelvin-type curvature effects at the solid–liquid interface [90]. Although this effect is generally modest, it may act synergistically with surface area enhancement to promote faster dissolution, particularly during the early stages where interfacial phenomena dominate. Taken together, the combined effects of increased surface area and curvature-driven changes in apparent saturation solubility are widely considered to contribute to the enhanced dissolution behavior of nanosized systems, providing a synergistic basis for dissolution improvement beyond that achievable by conventional micronization [90,91,92].

The presence of Poloxamer 188 further enhances dissolution through improved wettability and dispersion [93]. As an amphiphilic polymer, it adsorbs onto the hydrophobic surface of rivaroxaban nanoparticles, reducing interfacial tension and facilitating rapid penetration of the dissolution medium. In the SDS-containing medium, this effect is likely amplified through cooperative interactions between the adsorbed polymer layer and dissolved surfactant molecules, promoting efficient particle dispersion following tablet disintegration [94]. The slightly superior performance of F3 compared to F1 suggests a concentration-dependent effect of the stabilizer, with higher polymer content enabling more effective surface coverage and improved interfacial properties. Quantitatively, the higher Poloxamer 188 content in F3 resulted in a 6.2 percentage point increase in DE% at pH 4.5 (90.1% vs. 83.9%) and a 2.5 percentage point increase at pH 6.8 (60.8% vs. 58.3%).

In contrast, the physical mixtures (F2 and F4), despite containing the same excipients, showed markedly lower dissolution performance. At pH 4.5, F2 and F4 reached only ~72% dissolution at 120 min, with significantly lower DE% values (51.9% and 67.1%, respectively), and were statistically inferior to F1 and F3 (*p* < 0.001).

These results demonstrate that the dissolution advantage arises primarily from the nanosizing process—specifically, the increased surface area and enhanced drug–polymer interactions achieved during antisolvent precipitation—rather than from excipient composition alone.

While these effects dominate under sink-like conditions, their impact becomes more pronounced under solubility-limited conditions, where intrinsic solubility represents the primary constraint on drug release.

A markedly different and more discriminating pattern was observed in phosphate buffer at pH 6.8, where the absence of surfactant-imposed solubility-limited conditions more representative of the intestinal environment (Table 4). Under these non-sink conditions, all formulations exhibited lower but more clearly differentiated dissolution profiles.

At 120 min, the nanosuspension-based formulations (F1 and F3) achieved the highest extent of dissolution (66.2 ± 2.3% and 66.6 ± 1.2%, respectively), both significantly exceeding the commercial reference Xarelto^®^ 10 mg (53.9 ± 2.7%; *p* < 0.01). In contrast, the physical mixtures (F2 and F4) showed comparable performance to the reference (*p* > 0.10), while the micronized API exhibited substantially lower dissolution (28.7 ± 1.8% at 120 min).

The values for DE% over 180 min confirmed these differences, with F3 (60.8%) and F1 (58.3%) exceeding Xarelto^®^ (48.2%) by more than 10 percentage points, whereas F2 (45.3%) and F4 (52.4%) showed no meaningful improvement. The micronized API remained markedly inferior (DE = 21.9%).

Similarity factor analysis further supported these findings (Table 5). F1 (f_2_ = 53.1) and the physical mixtures (F2 and F4; f_2_ = 55.1–56.3) showed profiles similar to the reference, whereas F3 (f_2_ = 46.6) exhibited a non-similar profile, indicating a distinct dissolution profile with higher dissolution extent. All formulations showed non-similar (superior) profiles compared to the micronized API (f_2_ = 20.3–32.7).

At pH 6.8, F3 and F4 exhibited similar profiles (f_2_ = 52.0), whereas F1 and F2 did not (f_2_ = 43.5). At pH 4.5, neither pair showed similarity (f_2_ = 39.7 and 20.7, respectively), reflecting the reduced discriminative power of the surfactant-containing medium.

This pattern suggests that a higher Poloxamer 188 content contributes substantially to dissolution enhancement, partially compensating for the absence of nanosizing under solubility-limited conditions. However, at lower stabilizer levels, nanosizing remains the dominant factor governing dissolution performance.

The ability of the nanosuspension formulations to surpass the expected equilibrium solubility at this pH (reported to be approximately 5–6 µg/mL) [95] suggests the possible generation of a transient supersaturated state, although this was not directly measured in the present study. This behavior may be attributed to the combined effect of increased apparent solubility of nanocrystals and the presence of polymeric excipients capable of modulating precipitation kinetics [96,97]. In this context, HPMC may contribute to the stabilization of supersaturation by inhibiting nucleation and crystal growth, thereby prolonging the lifetime of dissolved drug species. Such behavior is consistent with the “spring–parachute” concept, in which rapid dissolution of nanosized particles generates supersaturation, while polymeric additives delay precipitation and maintain elevated drug concentrations in solution [96,98].

The statistically significant higher dissolution of F1 and F3 compared to Xarelto^®^ 10 mg at pH 6.8 (Δ = +12.3% and +12.7% at 120 min, *p* < 0.01) indicates that the antisolvent precipitation approach provides a measurable enhancement under solubility-limited conditions. This improvement is consistent with combined kinetic and interfacial effects associated with nanosizing and polymer stabilization.

To further characterize the release mechanism, dissolution data at pH 6.8 were fitted to standard kinetic models. The Korsmeyer–Peppas (KP) model with lag-time provided the best fit for all formulations (Table 6), with R^2^ values ranging from 0.9447 to 0.9996, whereas zero-order, first-order, and Higuchi models yielded substantially poorer fits for all formulations.

The estimated lag-times (3.10–4.94 min) were consistent with tablet disintegration and the initial wetting/dispersion phase preceding diffusion-controlled release. F3 exhibited the shortest lag-time, in agreement with its faster disintegration and improved wettability.

All formulations showed Fickian diffusion-controlled release (*n* ≤ 0.45). The improved dissolution performance of the nanosuspension formulations is reflected in their higher effective rate constants compared to the physical mixtures, consistent with increased surface area, improved wettability, and reduced crystallinity of RIV (as evidenced by XRD).

The physical mixtures displayed intermediate behavior, indicating that excipients such as Poloxamer 188 can enhance wettability to some extent, but are insufficient to reproduce the dissolution performance of nanosized systems. In contrast, the micronized RIV powder showed the lowest rate constant and the longest lag-time (*t*_lag_ = 14.07 min), indicating delayed wetting and slower dispersion in the dissolution medium. This behavior is consistent with its markedly reduced dissolution and confirms that conventional particle size reduction alone does not adequately overcome solubility limitations at neutral pH.

Taken together, the results obtained in the two dissolution media provide complementary insight into formulation performance. Under sink conditions, nanosuspensions primarily improve the rate of initial dissolution, whereas under non-sink conditions, they significantly enhance both the rate and extent of drug release. This distinction is particularly relevant from a biopharmaceutical perspective, as intestinal conditions are more closely approximated by the latter scenario.

Overall, the quantitative analysis confirms that the nanosuspension formulations provide a substantial and statistically significant improvement in dissolution performance. Under simulated intestinal conditions (pH 6.8), F3 and F1 achieved DE values of 60.8% and 58.3%, respectively, exceeding Xarelto^®^ 10 mg (48.2%) by more than 10 percentage points.

The f_2_ analysis further demonstrates that F3 shows a non-similar and superior profile relative to the commercial reference (f_2_ = 46.6), while F1 achieves similarity (f_2_ = 53.1) with directionally better dissolution.

Kinetic modeling indicated Fickian diffusion-controlled release for all formulations (Korsmeyer–Peppas model, *n* = 0.145–0.393, R^2^ = 0.945–0.9996), with the improved dissolution of nanosuspension systems reflected in their higher rate constants relative to the physical mixtures. F3 also showed the shortest lag-time (3.10 min), consistent with its faster onset of release. These results collectively demonstrate that F3 (RIV–P188–HPMC 1:2:1) is the most promising formulation for further development, combining superior dissolution extent, the fastest onset of release, and a DE advantage of over 12 percentage points relative to the marketed product under biorelevant conditions.

The novelty of the study lies in the development of a new method for formulating nanosuspensions, which allowed obtaining stable and efficient systems with improved pharmaceutical properties. The obtained results demonstrate the potential of this approach for optimizing the administration of poorly soluble drugs and support its applicability in the development of high-performance oral pharmaceutical forms.

## 4. Conclusions

Based on the obtained experimental results, corroborated with the physicochemical characterization and pharmaceutical performance of the developed formulations, the following conclusions can be formulated:

(i) Physico-chemical characterization by FTIR, XRD, SEM, and TGA revealed significant changes compared to the raw materials, confirming the formation of a nanosuspension-type system. FTIR analysis demonstrated compatibility between the components, without major chemical interactions. XRD results indicated the reduction or partial disappearance of the crystalline character of rivaroxaban, suggesting its amorphization. SEM images revealed uniform particles without well-defined crystals. TGA showed improved thermal stability due to interactions between the components.

(ii) Following the evaluation of the pharmacotechnical properties of the powders, it was found that only the dry nanosuspension formulations RIV-P188-HPMC (1:1:1) and RIV-P188-HPMC (1:2:1) presented adequate flow properties, allowing their compression. The formulation with a high HPMC content showed inadequate flow properties, which limited its processing into tablets, probably due to the hydrophilic character and increased cohesion of the particles.

(iii) The tablets obtained from the selected formulations demonstrated a superior dissolution profile compared to the commercial form of Rivaroxaban (Xarelto^®^ 10 mg), highlighting the effectiveness of the nanosuspension in increasing the solubility and release rate. Among them, the formulation with a higher Poloxamer 188 content presented the best dissolution performance, due to its ability to improve particle wetting and dispersion.

In conclusion, optimizing the molar ratio of excipients significantly influences both technological properties and biopharmaceutical performance, with the poloxamer-rich formulation being the most promising for further development.

## Figures and Tables

**Table 1:** The formulations of the oral tablets.

Ingredients	Quantity mg/Tablet						Role in Formulation
	F1	F2	F3	F4	F5	F6	
RIV-P188-HPMC (1:1:1 dry nanosuspension)	30	-	-	-	-	-	Active ingredient
RIV-P188-HPMC (1:1:1 physical mixture)	-	30	-	-	-	-	Active ingredient
RIV-P188-HPMC (1:2:1 dry nanosuspension)	-	-	40	-	-	-	Active ingredient
RIV-P188-HPMC (1:2:1 physical mixture)	-	-	-	40	-	-	Active ingredient
RIV-P188-HPMC (1:1:2 dry nanosuspension)	-	-	-	-	40	-	Active ingredient
RIV-P188-HPMC (1:1:2 physical mixture)	-	-	-	-	-	40	Active ingredient
Avicel^®^ PH 102—microcrystalline cellulose	83	83	78	78	78	78	Filler Binder
Flowlac^®^ 100—spray-dried lactose	83	83	78	78	78	78	Filler Binder
EXPLOTAB^®^—Sodium starch glycolate	2	2	2	2	2	2	Superdisintegrant
LIGAMED^®^ MF-2-V—Magnesium stearate	2	2	2	2	2	2	Glidant
TOTAL	200	200	200	200	200	200	

**Table 2:** Main FTIR peaks shift analysis.

Functional Group	υ(N-H)	C-H Stretching Vibration	υ(C=O)	β(N-H)	C-O-C Stretching Vibration	C-H Deformation Vibration
RIV (cm^−1^)	3358.47	-	1669.11	1645.49	-	-
P188 (cm^−1^)	-	2884.55	-	-	1105.9	842.2
RIV-P188-HPMC (1:1:1) (cm^−1^)	3356.08	2891.00	1668.15	1645.97	1104.5	841.7
RIV-P188-HPMC (1:2:1) (cm^−1^)	3361.85	2880.21	1670.10	1647.90	1103.1	842.7
RIV-P188-HPMC (1:1:2) (cm^−1^)	3353.17	2886.96	1669.11	1648.38	1106.4	843.2

**Table 3:** The thermal parameters obtained from the TGA of the studied compounds.

Compound	Peak Temperature (°C) and Mass Loss (%) (1st Step)	Temperature Range, Peak Temperature (°C) and Mass Loss (%) (2nd Step)	Temperature Range, Peak Temperature (°C) and Mass Loss (%) (3rd Step)	Residue (%) at 700 °C
RIV	*T*_DTA_ = 231.9 °C (melting process)	270–700 °C *T*_DTA_ = 316.6 °C 65.5%	-	34.5%
HPMC	*T*_DTA_ = 76.6 °C (dehydration process) Below 114 °C/7.43%	190–365 °C *T*_DTA_ = 324 °C 78%	365–650 °C *T*_DTA_ = 461 °C 14.57%	No residue
P188	*T*_DTA_ = 62 °C (melting process)	240–700 °C *T*_DTG_ = 381.8 °C 100%	-	No residue
RIV-P188-HPMC (1:1:1)	*T*_DTA_ = 57 °C (melting process of P188) *T*_DTA_ = 224.6 °C (melting process of RIV)	250–450 °C *T*_DTA_ = 337.1 °C *T*_DTA_ = 384.6 °C 74.4%	450–700 °C 11.7%	13.9%
RIV-P188-HPMC (1:1:2)	Below 60 °C (Dehydration process) 4.4%	100–450 °C *T*_DTG_ = 179.4 °C *T*_DTA_ = 344.4 °C 87.6%	450–700 °C 8.0%	No residue
RIV-P188-HPMC (1:2:1)	*T*_DTA_ = 57.7 °C (melting process of P188) *T*_DTA_ = 232.7 °C (melting process of RIV, with decomposition)	190–400 °C *T*_DTG_ = 335.2 °C *T*_DTA_ = 382.7 °C 80.7%	400–700 °C 19.3%	No residue

**Table 4:** Comparative dissolution performance of RIV formulations in pH 4.5 (acetate buffer with 0.2% SDS) and pH 6.8 (phosphate buffer). For pH 4.5, Q15, Q30, and Q120 were selected to capture early and plateau phases under sink conditions, while for pH 6.8, Q30, Q60, and Q180 represent the early, discriminative, and late stages of dissolution under non-sink conditions.

Product	Medium: pH 4.5 Acetate Buffer with 0.2% SDS				Medium: pH 6.8 Phosphate Buffer			
	Q15 (%)	Q30 (%)	Q120 (%)	DE%	Q30 (%)	Q60 (%)	Q180 (%)	DE%
F1	59.6 ± 10.4	80.0 ± 11.3	104.4 ± 6.3	83.9 ± 2.8	49.0 ± 5.9	60.8 ± 3.1	67.9 ± 5.9	59.0 ± 0.9
F2	18.5 ± 2.6	34.2 ± 3.3	72.4 ± 3.5	51.9 ± 2.1	32.9 ± 2.3	45.7 ± 1.8	58.3 ± 3.4	45.5 ± 0.3
F3	67.6 ± 1.3	85.7 ± 5.9	106.5 ± 5.3	90.1 ± 1.9	57.7 ± 3.7	63.8 ± 1.6	67.8 ± 1.9	61.7 ± 1.1
F4	61.7 ± 1.6	63.5 ± 2.2	73.0 ± 1.0	67.1 ± 1.5	45.6 ± 1.5	53.9 ± 2.1	57.7 ± 2.1	52.4 ± 1.2
RIV powder	54.1 ± 4.0	65.8 ± 4.3	69.8 ± 5.3	63.1 ± 2.3	11.8 ± 2.0	22.6 ± 2.1	34.4 ± 2.4	21.9 ± 0.8
Xarelto^®^ 10 mg	85.0 ± 1.6	89.9 ± 0.3	93.8 ± 2.8	86.8 ± 1.6	45.8 ± 2.0	50.9 ± 2.3	54.3 ± 1.7	48.2 ± 1.2

Data are expressed as mean ± standard deviation (SD) (*n* = 3). Q values represent the cumulative percentage of drug dissolved at the indicated time points.

**Table 5:** Similarity factor (f_2_) values for pairwise comparison of dissolution profiles in pH 4.5 and pH 6.8 media. An f_2_ value ≥ 50 indicates similarity between profiles, while values < 50 indicate non-similar dissolution behavior. N/A indicates that f_2_ was not calculated due to rapid dissolution (>85% within 15 min).

Comparison	f_2_ (pH 4.5)	f_2_ (pH 6.8)
F1 vs. Xarelto^®^ 10 mg	N/A	53.1
F3 vs. Xarelto^®^ 10 mg	N/A	46.6
F2 vs. Xarelto^®^ 10 mg	N/A	55.1
F4 vs. Xarelto^®^ 10 mg	N/A	56.3
F1 vs. F3	51.8	65.5
F1 vs. F2	20.7	43.5
F3 vs. F4	39.7	52.0
F1 vs. RIV	47.4	22.5
F3 vs. RIV	41.9	20.3
F2 vs. RIV	30.8	32.7
F4 vs. RIV	55.2	23.9

**Table 6:** Kinetic parameters obtained by fitting dissolution data (pH 6.8) to the Korsmeyer–Peppas. k_KP_ is the apparent rate constant, *n* is the release exponent, t_lag_ is the lag-time, and R^2^ is the coefficient of determination.

Product	k_KP_ (%·min^−n^)	*n*	t_lag_ (min)	R^2^
F1	17.55	0.32	4.52	0.9980
F2	11.82	0.32	4.35	0.9784
F3	15.86	0.39	3.10	0.9996
F4	12.10	0.15	3.18	0.9634
RIV powder	4.06	0.41	14.07	0.9992
Xarelto^®^ 10 mg	10.95	0.17	4.94	0.9457

## Data Availability

The original contributions presented in this study are included in the article. Further inquiries can be directed to the corresponding author.
